# National human footprint maps for Peru and Ecuador

**DOI:** 10.1038/s41597-025-06301-0

**Published:** 2025-12-24

**Authors:** Jose Aragon-Osejo, Lenin Beltrán, Juan Iglesias, Hólger Zambrano, Daniel Borja, Carlos Oñate, Francisco Simbaña, Freddy Valencia, Karen Rodríguez De la Vera, Luis Poveda, Fernando Proaño, Lorena Parra, María Chacón, William Llactayo, Tatiana Pequeño Saco, Walter Huamani, German Marchand, Raúl Tinoco, Luis Quispe, Alexs Arana, Anne Lucy Stilger Virnig, Patricia Huerta, Karla Jiménez, Pedro Tipula Tipula, Susana Rodríguez, Rodrigo Sierra, Andrew J. Hansen, Oscar Venter

**Affiliations:** 1 Natural Resources and Environmental Studies Institute, University of Northern British Columbia, Prince George, BC Canada; 2 Ministerio del Ambiente, Agua y Transición Ecológica de Ecuador (MAATE), Quito, Ecuador; 3 Ministerio del Ambiente del Perú (MINAM), Lima, Perú; 4 Universidad Nacional Federico Villarreal, Lima, Perú; 5 Rainforest Alliance (RA), Lima, Perú; 6 Servicio Nacional Forestal y de Fauna Silvestre (SERFOR), Lima, Perú; 7 United Nations Development Programme, New York, NY USA; 8 Programa de las Naciones Unidas del Desarrollo - Perú (PNUD), Lima, Perú; 9 Independent Contributor, Quito, Ecuador; 10 Independent Contributor, Lima, Perú; 11 Instituto de Investigación de Recursos Biológicos Alexander von Humboldt, Bogotá, Colombia; 12 GeoIS, Austin, TX USA; 13 Professor Emeritus of Ecology, Montana State University, Bozeman, MT USA

**Keywords:** Environmental impact, Ecological modelling, Conservation biology

## Abstract

Human Footprint (HF) maps score human pressures based on their influence and integrate them into a single spatial index to assess the naturalness of ecosystems. We produced a historical series of national HF maps for Peru and Ecuador for Sustainable Development Goal 15 (SDG15) reporting. These maps integrate pressures from built environments, land use/land cover (LULC—agriculture, pasture, tree plantations), roads and railways, population density, electrical infrastructure, oil and gas infrastructure, and mining. The dataset includes HF maps and individual pressure maps for Peru from 2012 to 2021, as well as for Ecuador for the years 2014, 2016, 2018, 2020, and 2022. These maps support the analysis of spatiotemporal patterns of human influence at national and subnational levels, enabling biodiversity monitoring, modelling, and conservation in these highly biodiverse countries.

## Background & Summary

The Global Indicator Framework (GIF) consists of standardized indicators used to measure progress on the Sustainable Development Goals (SDGs) outlined in the 2030 Agenda for Sustainable Development^1–3^. In addition to the official GIF indicators^4^, complementary indicators^2^ can provide important information on progress towards SDGs. Examples related to SDG15 include analyses of human influence on ecosystems and the use of riparian and core forest metrics, which evaluate aquatic system protection and landscape fragmentation^5^. We produced National Human Footprint (HF) maps as datasets for complementary indicators for SDG15 –Life on Land– for Peru and Ecuador to focus on forest quality and assess ecosystem naturalness^5–8^.

We adapted global HF methods^6^ to produce HF maps for Peru and Ecuador, aligning them with GIF requirements^9^. We produced a historical series at 30 m resolution—suitable for national and subnational projects—enabling analysis of spatiotemporal patterns of human influence. These maps cover the entirety of the terrestrial land area of both countries, including all ecosystems, but exclude natural inland waters and Ecuador’s Galápagos Islands. They integrate spatial proxies of human pressures—built environments, land use/land cover (LULC—agriculture, pasture, tree plantations), roads and railways, population density, electrical infrastructure, oil and gas infrastructure, and mining—into a single index. Validation of the HF was conducted through visual interpretation of high-resolution imagery from World Imagery^6,10^ (<0.5 m resolution), resulting in a strong overall performance. The HF maps for SDG15 reporting (“SDG15 HF”) sought to optimize accuracy, resolution, scale, and completeness, applying a non-restrictive input-selection strategy, i.e., favouring the most beneficial dataset when multiple options were available. This strategy outperforms other GIF-relevant HF approaches (Official- and Multitemporal-HF) for SDG reporting^9^ (see section Input selection strategies).

HF maps have strong potential for SDG15 reporting, which aims to conserve terrestrial ecosystems and halt biodiversity loss^11^. Intact landscapes—those with greater naturalness—support key attributes for biodiversity conservation^12,13^, though areas with higher human influence can still contribute^14,15^. Under the assumption that as human influence increases, ecosystem naturalness and its attributes decline^6,8,16^, HF maps can support SDG15 by representing human influence as a continuum ranging from absent to high.

The HF maps have been widely used in global and regional biodiversity studies, supporting research on intact terrestrial ecosystems and wilderness areas^6,17,18^, species extinction risk^19–21^, protected areas’ effectiveness^22^, plant diversity^23^, species’ tolerances to human pressures^24^, habitat suitability^14^, and the occurrence of vector-borne disease and virus transmission^25,26^. Future similar studies in Peru and Ecuador can incorporate a historical HF series with a resolution suitable for national and subnational use. Additionally, the scripts allow flexible adaptation of input datasets, formats (vector, raster, categorical, continuous), scoring schemes, and pressure organization, supporting the development of national HF maps beyond Peru and Ecuador.

## Methods

### Methods overview

**Fig. 1:**
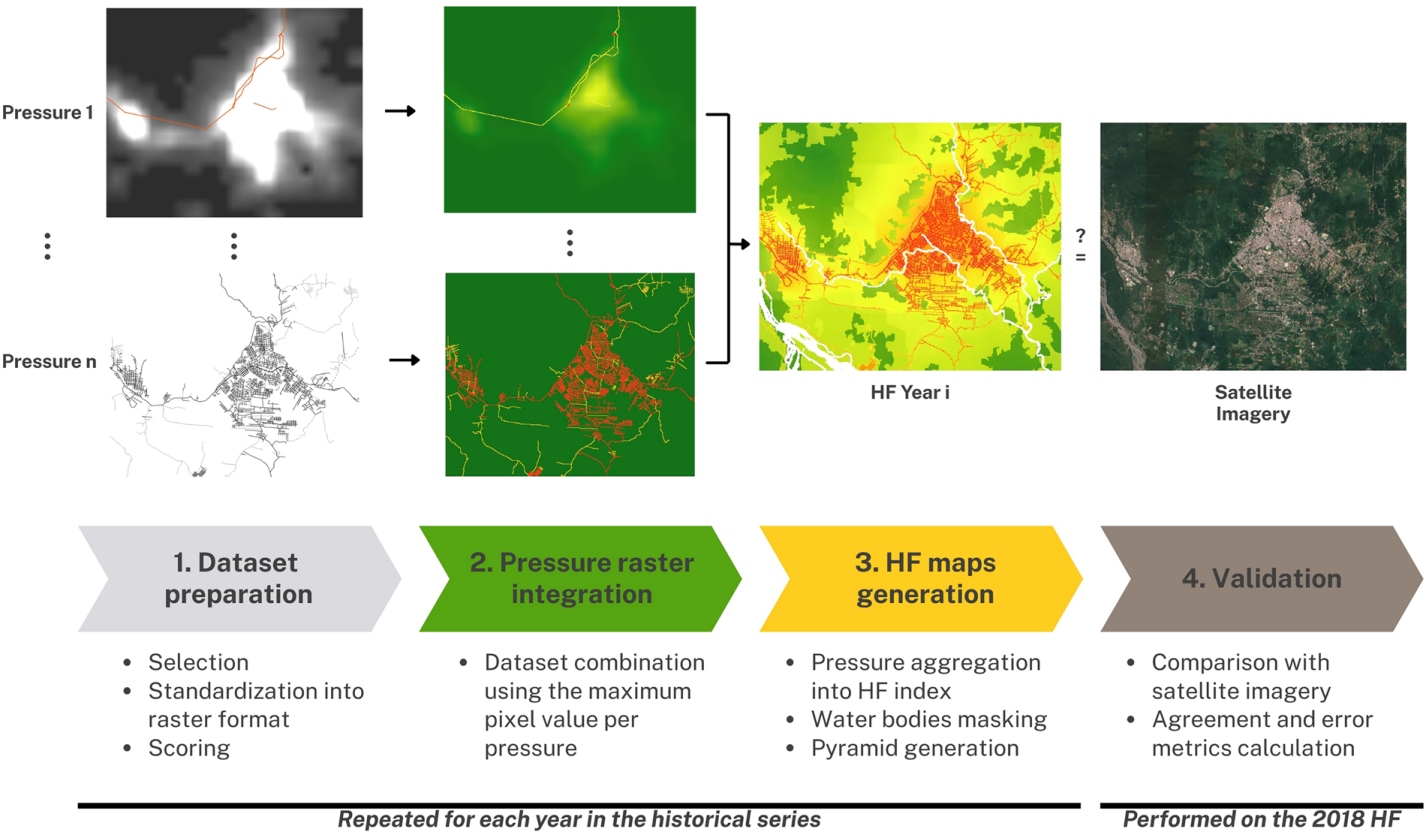
Peru and Ecuador HF maps generation workflow. (1) Datasets were collected, unsuitable ones discarded, and the remainder selected through an input-selection strategy. All were standardized into raster format with a common projection and pixel size, then scored for human influence. (2) Scored rasters were combined by pressure using the maximum pixel value to produce pressure rasters. (3) Pressure rasters were aggregated to generate HF index maps, water bodies were masked out, and pyramids were generated for efficient handling. Steps 1–3 were repeated for each year in the historical series. (4) Validation was conducted by comparing the 2018 HF raster with satellite imagery.

We produced HF maps based on the global HF methods^6^, as shown in Fig. 1. HF maps score spatial input datasets of human pressures according to their influence and integrate them into a unique spatial index. We included the pressures of built environments, land cover/land use (agriculture, pasture, tree plantations), roads and railways, population density, electrical infrastructure, oil and gas infrastructure, and mining. Input datasets of pressures were prepared and scored for their influence individually in a raster format. Then, they were combined by maximum pixel value when multiple input datasets pertained to the same pressure but represented different features (e.g., primary, secondary, country roads and railways within the pressure of roads and railways). Scored pressures were aggregated to produce the HF maps. Then, water bodies such as rivers and lakes were masked, values were rounded to 2 decimals if needed, and pyramids were created. Finally, validation metrics—including Cohen’s kappa statistic, root mean squared error, and R²—were calculated for the 2018 HF.

We distinguished between direct pressures, occurring at the location indicated by input datasets, and indirect or inferred pressures, likely to happen even though no datasets represented them^23,27^. In our study, all pressures are direct except for “Indirect pressures,” which are represented as a proxy based on accessibility. We also distinguished input datasets as either multitemporal, with multiple updates since 2012, or static, having only one update within the same period.

Visible Infrared Imaging Radiometer Suite (VIIRS) nighttime lights^28^ and LULC layers dictated the number of updates. VIIRS provided global updates from 2012 to 2022. In Ecuador, national land use layers^29,30^ were mandated by the Ministry of Environment, Water and Ecological Transition (MAATE), resulting in updates for 2014, 2016, 2018, 2020, and 2022. In Peru, MapBiomas project^31^ layers were used to produce annual maps from 2012 through 2021.

The HF maps for Peru and Ecuador were produced using identical methods and scripts, but the processes were independent for each country and used different input datasets. We used projection WGS84 UTM 18S for Peru and 17S for Ecuador, maintaining a 30-meter resolution. We used bilinear resampling for continuous values and the mode for categorical variables.

We used Anaconda^32^ and open libraries to avoid license issues or extra costs. This approach proved feasible, yet we encountered installation difficulties on users’ desktops, and input folders (~50GB) were difficult to transfer.

### Input data collection

This dataset was produced within the “NASA Life on Land” project (2019–2023), a collaboration between national Ministries of Environment, the United Nations Development Programme, and external academic producers to co-produce datasets and complementary indicators for SDG15, specifically to monitor forest quality^5^. During its first stage (~2020), the project collected nationally produced layers representing pressures on terrestrial ecosystems in Peru and Ecuador. Requests, accompanied by an explanation of the project’s goals and how the datasets would be used, were sent out to potential sources by local ministry counterparts to ensure an official level. Additionally, we collected datasets from cumulative pressure mapping studies. We filtered out layers that were not spatially explicit or lacked national coverage. In Ecuador, national land use layers were mandated by the MAATE^29,30^.

The collection was heterogeneous in temporal coverage, source, number of updates, scale/resolution, format, and quantity of datasets per pressure. Spatial inputs were assessed by comparing them with their attributes and documentation against other layers and high-resolution imagery. This resulted in an overall understanding of their limitations, and which inputs provided higher achievement of characteristics expected by the GIF^9^.

### Input selection strategies

**Fig. 2:**
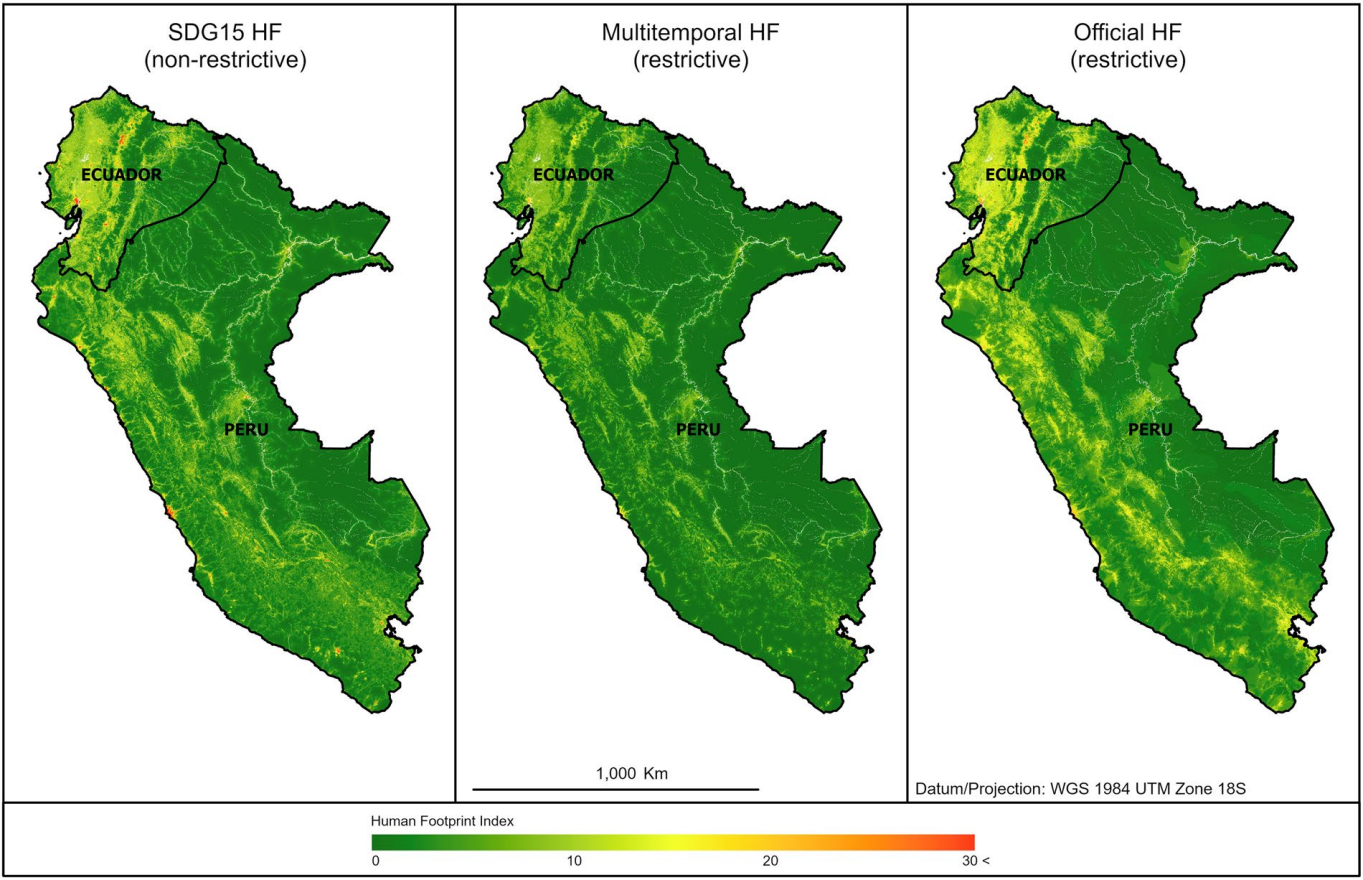
SDG15, Multitemporal, and Official HF maps for Peru and Ecuador in 2018. The Official and Multitemporal versions followed restrictive input selection strategies, while the SDG15 version adopted a non-restrictive approach. HF values are presented on a 0–30 scale to ensure index comparability across versions. Although spatial patterns are generally similar, differences in colour intensity, particularly in the yellow tones, are noticeable. Each HF map version was created independently for Peru and Ecuador. HF maps were created in WGS 1984 UTM Zone 18S for Peru and Zone 17S for Ecuador.

We produced three GIF-related versions of the HF maps for Peru and Ecuador: SDG15, Official, and Multitemporal (Fig. 2). For the SDG15 HF, the approach was “non-restrictive,” i.e., if no dataset for a specific pressure met all expected characteristics, the best option available was used. It was expected to optimize accuracy, resolution, scale, and completeness. For instance, oil wells did not exist as a multitemporal layer, and nighttime light rasters had a coarser resolution than the rest of the input datasets, but were still used. The SDG15 HF version was recommended as the National HF maps in Peru and Ecuador for SDG15 reporting. The input selection strategy for the Official and Multitemporal HFs targeted specific characteristics. The Official HF followed the expectation of the UN Statistical Commission^2,33^ and of the Life on Land project to use only nationally produced datasets. The Multitemporal HF followed an “all multitemporal inputs” approach designed for indicator analysis over time. This approach is recurrent in the literature on cumulative pressure maps^34–36^. These maps were “restrictive” and expected to be “less complete” (i.e. to integrate fewer input datasets) than the SDG15 version.

### Human footprint scoring template

Our HF scoring template for datasets is based on the global HF^6,7^ for general comparability, with scores ranging from 0 to a dataset-dependent maximum never exceeding 10. A score of 4 or above signals a loss of the original ecosystem. Datasets’ maximums were evaluated using the following criteria, ordered by importance:

1. Loss of original ecosystem cover (i.e., land cover change),
2. The imperviousness of surfaces, such as urban areas, roads, and infrastructure. Impervious surfaces block water infiltration, prevent vegetation growth and indicate long-term or irreversible changes^37,38^,
3. Habitat viability, indicating the potential to support biodiversity or allow for passage, and,
4. Pollution risk.

Each criterion received an importance value (“CI” in Table 1). The sum of all CIs is 10. All datasets received a qualification between 0 and 1 for each criterion, and the score for each dataset is the sum of all criterion values, each multiplied by its respective importance. Please refer to this table for the dataset scoring used in the following section.

**Table 1:** Dataset scores calculated from the individual criterion values.

Pressure: Datasets*	Original cover loss	Impervious surface	Not viable habitat /passage	Pollution risk	Dataset maximum score
	CI = 4**	CI = 3	CI = 2	CI = 1	
**BE: Urban areas**	1	1	1	1	**10.00**
**PD: Densely populated areas EI: Densely illuminated areas**	1	1	1	1	**10.00**
**OG: Wells, depots, refineries, stations, filling stations**	1	1	1	1	**10.00**
**EI: Power plants, dams, substations, power towers, wind turbines**	1	1	1	0	**9.00**
**RR: Primary roads, railways, BE: Runways**	1	1	0.75	0.25	**8.75**
**EI: Solar, power and hydroelectric plants**	1	0.75	1	0	**8.25**
**RR: Secondary roads**	1	1	0.5	0.25	**8.25**
**BE: Settlements, ports, sparse houses**	1	0.75	0.5	0.25	**7.50**
**M: Mining areas**	1	0.25	0.75	1	**7.25**
**LU: Artificial water bodies**	1	0	1	0	**6.00**
**RR: Country roads**	1	0.5	0.25	0	**6.00**
**LU: Pasture**	1	0	0.75	0	**5.50**
**LU: Agriculture**	1	0	0.5	0.25	**5.25**
**OG: Pipelines**	1	0	0.5	0.25	**5.25**
**EI: Transmission and power lines**	1	0	0.5	0	**5.00**
**LU: Tree plantations**	1	0	0.25	0	**4.50**
**RR: Trails**	0	0.25	0.25	0	**1.25**

*Pressure corresponding to each dataset. BE: Built environments, LU: Land use/land cover, PD: Population density, RR: Roads and railways, EI: Electrical infrastructure, OG: Oil and gas infrastructure, M: Mining.

**CI: Criterion importance.

Maximum scores were used to convert the original data into raster representations of human influence. To apply the scores above, we considered the following scoring cases:

1. Score assigned to all pixels at the direct location: Used for layers organized in categories, for example, from LULC, and linear features such as roads.
2. Exponential decay scoring of distances: We assumed that influence is more intense at the source surroundings, then rapidly decays, and still likely exists up to a distance. This is the method used in the global HF for the indirect pressure.
3. Logarithmic scoring: We assumed that small values of pressure can still exert a significant influence. This is the method used in the global HF for population density.
4. Linear scoring: We assumed that the influence increases or decreases in direct proportion to the pressure, following a linear transformation. This is a default assumption until more knowledge exists on better scoring approaches^16^.
5. Points as approximate locations: A score was assigned to the pixel at the point’s location, and an exponentially decaying score was assigned to its surroundings. This strategy allows for representing the existing influence of a point feature while controlling the lack of spatial accuracy of the dataset.
6. Points at actual locations: We applied the score to the pixel at the point’s location and to surrounding pixels within a distance determined after satellite imagery exploration, to better represent the size of the features. This adjustment was used for features larger than the 30*30 m pixel size, such as oil refineries and stations.

The following sections describe the pressures and datasets used, as well as the scoring procedures applied.

### Pressures’ input datasets and scoring

For the input datasets below, please check the corresponding references for access links or indication that a request is necessary (“by request”). Since most of the data was collected in 2020, some links may no longer be functional, and certain datasets might no longer be openly available. When no currently active alternative link was found, the reference includes the note “Inactive link”. For these cases, as well as for input datasets available upon request, we recommend contacting the respective institutions directly for access. In Ecuador, openly available basic cartography does not include reserved zones, for which users need to submit a formal request^39^.

We note that OpenStreetMap^40^ (OSM) was used in this study. Despite issues with scale heterogeneity and attribute assignment^41,42^, we considered that OSM demonstrated superior accuracy in representing ground features related to roads and railways, electrical infrastructure and mining compared to alternative datasets. Our choice to use OSM aligns with other cumulative mapping studies^6,43,44^.

#### Built environments

In the Built Environment pressure, we included areas of human habitation such as urban zones, towns, villages, and sparsely populated areas like scattered houses. Urban areas are highly modified environments, usually detectable from remote sensing. They lack significant ecosystem services or species habitats and can feature elevated temperatures, altered rainfall patterns, and increased noise and light levels. These effects can affect reproduction cycles, potentially leading to declines in species richness, abundance, diversity, and evenness and phenotype differences from rural habitats^45–47^. These changes may happen within smaller settlements like towns and villages, though at a lower level. While scattered houses might not induce similar changes, they are indicators of human presence.

For the SDG15 HF, we used urban zones from multitemporal LULC layers, i.e., MapBiomas Perú^31^ and land use layers from MAATE^29,30^, and Meta’s static sparse houses and buildings^48^ all at a 30 m resolution. Urban zones capture most of the infrastructure inside their boundaries, although features like urban parks, ports and runways might not be considered. We complemented urban zones and sparse houses with runways and ports from the Military Cartographic Institute in Ecuador^39^ (IGM) and the Ministry of Environment in Peru (MINAM)^49,50^ available as point features. For the Official HF, urban zones were complemented by settlements (towns and villages), represented as point features from IGM, MINAM and the Ministry of Culture in Peru^51^. Point features were used when polygons were not available.

We assigned urban areas a score of 10, runways 8.75 and sparse houses pixels a score of 7.5. Settlement and port point layers were given a score of 7.5, with indirect pressure around them decaying exponentially from 4 to a maximum distance of 100 m.

#### Land cover/land use (agriculture, pastures and tree plantations)

The LULC pressure layer predominantly involved agricultural activities, pastures, tree plantations, and oil palm, implying extensive human alteration of the landscape. These land covers and uses often result in losing original natural cover, leading to ecosystem fragmentation, degradation, and habitat destruction^52,53^. Agriculture and pasture have been reported to be significant drivers of deforestation^54–56^ and increasing in Peru and Ecuador^57–59^. Although also replacing natural land cover, forestry trees and oil palm plantations might provide a viable habitat^15,60^.

We included agriculture, pasture, forestry, and oil palm plantations from 30 m resolution multitemporal LULC layers^29,31^ for the SDG15 HF. Peru’s Official HF included a 2018 official static agricultural census layer from the Ministry of Agrarian Development and Irrigation (MIDAGRI)^61^, and we also included a 2017 official static layer of tree plantations from the Ministry of Agriculture and Livestock in Ecuador^62^.

In Peru, pasture received a score of 5.5, while agriculture received a score of 5.25. In Ecuador, inconsistencies across updates affected pasture, crops, oil palm plantations, agricultural mosaic categories and artificial water bodies, leading us to assign a unique score of 5.25. Tree plantations in Peru and Ecuador were given a score of 4.5.

#### Population density

Population density significantly determines the intensity of human pressure on the landscape. In Peru and Ecuador, official census data serve as the primary source for population density information. These datasets result from comprehensive field surveys conducted approximately every ten years. While these datasets might have high accuracy, census data suffers from spatial limitations, as it relies on census units that can be sizable and susceptible to the Modifiable Areal Unit Problem^63^. Satellite imagery and disaggregation methods have been used to address these limitations, often employing artificial intelligence and machine learning-based dasymetric redistribution. These techniques enable a shift from census data estimations, pinpointing households and populated areas at pixel levels^48,64–66^. This approach might employ interpolation and extrapolation to generate estimates for the years other than the official census dates, enhancing temporal coverage^65,67^. While official censuses are necessary for national projects, these techniques offer advantages for spatial studies.

For the SDG15 HF, we used WorldPop^67^. Despite the dataset spanning from 2000 to 2020, we considered WorldPop layers static, using the base census years 2007 for Peru and 2010 for Ecuador. Our decision was based on reviewing the extrapolated years that indicated zones of modelled population growth inconsistent with our knowledge. For the Official HF, we obtained official data from National Statistics Institutes, i.e., INEI in Peru^68^ and INEC in Ecuador^69^, capturing information at the census unit level.

We logarithmically scaled scores to highlight the influence that even small population densities might have on the landscape^7^. Regarding WorldPop, the population counts surpassing 70 individuals per pixel received a score of 10, while the rest were scored using Eq. 1:

1 $${\rm{Score}}=5.41\ast \log ({\rm{population\; count}}+1)$$

The 70 individuals per pixel threshold approximates the upper values found within densely populated cities in both countries. The coefficient of 5.41 in the equation ensured that values near 70 receive an approximate score of 10. Similarly, we scored population density from national census layers derived from official datasets using Eq. 2:

2 $${\rm{Score}}=2.5\ast \log ({\rm{population\; density}}+1)$$

#### Indirect pressures

We developed a new approach to assess indirect pressures, i.e., pressures accompanying human presence that cannot be observed or represented from existing datasets^27^. These include activities that can alter the original ecosystem composition without removing it, such as selective logging and hunting^70–73^.

We estimated indirect pressures from the perspective of human accessibility and the effects of remoteness on natural areas^74^. The premise rested on the idea that individuals consistently exert more influence on areas close to their home bases than at a distance. This approach concentrated on the consistent impact of routine activities while disregarding more sporadic events. We followed Weiss *et al*.^74^ and Watmough *et al*.^75^ to calculate a proximity surface to human habitation from the built environment layer by creating a cost allocation raster and performing a least cost path analysis.

For creating a cost allocation raster, the cost within the allocation raster corresponded to the time required to traverse a 30 m pixel horizontally or vertically. Speeds were modelled from tracking data in Ecuador^76^. Speeds depended on the transportation modes and the environmental characteristics. We used an order of precedence to prioritize higher speeds on the cost allocation raster, as shown in Table 2. Built environment pixels were assigned a null cost (i.e., a speed of 0 km/h).

**Table 2:** Precedence order and speed calculation according to the transportation means and environmental features.

Transportation means and environmental characteristics in order of precedence	Categories	Average speed (km/h)				
1. Roads	*Primary*	60				
	*Secondary*	40				
	*Tertiary (country roads)*	30				
2. Coastline (navigation)		20				
3. Waterways (rivers and lakes)		*slope (%)*				
	Elevation*	*<5*	*5–10*	*10–15*	*15–25*	*>25*
	*<450*	15	7.5	3.8	1.9	1.4
	*450–700*	7.5	3.9	2.7	1.9	1.4
	*700–1800*	3.8	2.7	2.0	1.7	1.4
	*1800–2800*	1.9	1.9	1.7	1.4	1.3
	*>2800*	1.4	1.4	1.4	1.3	1.2
4. Agriculture (walking)		10.560326*slope(%)^−0.199553^				
5. Forest, shrubs, and herbaceous vegetation (walking)	*Non-flooded*	−0.975931*ln(slope (%)) + 6.761258				
	*Flooded*	$\frac{-0.975931\ast \mathrm{ln}({\rm{slope}}\left( \% \right))+6.761258}{2}$				

*Metres above sea level.

For the least cost path analysis, we calculated the least cost path from each pixel to inhabited areas in the Built environments layer. To mitigate instances where sparse houses or buildings were too small for habitation, we only considered clusters of at least two pixels as sources for accessibility. For Peru, processing exceeded the available RAM capacity. We partitioned the country into four polygons with over 100 km wide overlaps. The least cost analysis for these polygons was conducted separately and then merged using the minimum pixel value, keeping the shortest distance from Built environments.

Time values from the least cost path raster were scored, decaying exponentially from a human influence score of 4. The accessibility analysis used multitemporal data of built environments and agricultural areas and static data related to roads and rivers, resulting in a partially multitemporal analysis.

The maximum time allowed was 4 hours. This travel time limit was based on a 12-hour daylight routine, encompassing travel to work, work hours, and return trips. Our assumption considered a minimum of 4 hours daily on work, setting the farthest travel distance at 4 hours away. This time limit does not imply that influence ceases beyond this point, but approximates a limit to routine human influence. This limit aligned with findings by Banerjee *et al*. in India^77^, where higher travel time expenditures ranged from approximately 2 to 10 hours. The authors argued that these travel patterns are context-dependent and vary significantly based on transportation modes.

This method of calculating indirect pressures represents an improvement over existing approaches. The global HF methodology^6,7^ considered indirect pressures associated with roads and rivers, employing an exponentially decaying function from centerlines. While the distance from built environments was partially considered for waterways, distance on roads from built environments, terrain variations and diverse walking speeds were not factored into the analysis. Similarly, Colombia’s Legacy-adjusted Human Footprint Index^34^ scored indirect pressures by categorizing buffers from Euclidean distances from roads and settlements into ranges. These methods allowed for estimating indirect pressures but are based on broad assumptions. Our approach addressed important transportation conditions, providing a more realistic representation of mobility behaviour. Additionally, the 30-meter resolution retained road sinuosity and topographical relief, ensuring precise estimations.

Our approach has limitations, as highlighted by Weiss *et al*.^74,78^ and Watmough *et al*.^75^. We lacked sufficient information to account for the directional impact of slopes, diverse transportation preferences or destinations, the influence of ports and stations, road management^70,79^, barriers to mobility, seasonality^80^, or the time required to transition between different modes of transportation. In addition to reported limitations, the layer representing houses and buildings requires refinement, and we did not consider population density of built environments.

We used an exponentially decaying function to represent indirect pressures, like other studies^6,27^. It should be noted that the assumption of decaying human influence contrasts with Weiss *et al*.^74^ of a deforestation peak between 1 and 5 hours from cities. However, our study focuses on general human influence, encompassing various aspects beyond deforestation and considering all sizes of human habitation, not exclusively cities.

Lastly, the raster accessibility model has been reported to be highly sensitive to speed variations^81^. It will be important to improve speed models at the national level to increase the accuracy of our approach.

#### Roads and railways

Roads fragment ecosystems, introduce edge effects, hamper dispersal, alter microclimate, and lead to death by collision. They contribute to soil erosion and the dissemination of invasive species. Beyond these direct effects, roads facilitate pollution, stimulate land clearing, illegal logging, hunting, and the trade of forest products^82–85^. Railways can have similar impacts while affecting ecosystems differently^86^.

We used OSM^40^ for road data for the SDG15 HF in Peru and Ecuador due to its higher completeness level than official sources (IGM in Ecuador and Ministry of Transport and Communications in Peru^39,87^). Road types used in this study and corresponding categories in the OSM dataset are found in Table 3.

**Table 3:** OSM tags of Road types.

Dataset	OSM key	OSM values
Primary	highway	living_street, motorway, motorway_link, primary, primary_link, residential, service, trunk, trunk_link
Secondary	highway	secondary, secondary_link
Country roads	highway	bridleway, tertiary, tertiary_link, track, track_grade1, track_grade2, track_grade3, track_grade4, track_grade5, unclassified, unknown
Trail	highway	footway, path, cycleway, pedestrian, steps

New roads are uploaded to OSM based on data availability, which might not accurately reflect on-the-ground changes. Addition dates, instead of creation dates, could mislead trends in indicators calculated from an OSM-based dataset. Therefore, we did not classify this source as multitemporal, although it remains an important area for improvement.

We chose railway data sources based on completeness. In Peru, we relied on OSM, while in Ecuador, we utilized IGM’s railway data. Despite the multitemporal nature of official roads in Peru^87^, we considered them static since they lacked updates beyond 2018.

We assigned a score of 8.75 to primary roads and railways, 8.25 to secondary roads, and 6 to country roads. Additionally, OSM’s trail data was assigned a score of 1.25.

#### Electrical infrastructure

The electrical infrastructure pressure layer included electric lights, electricity production, and distribution infrastructure.

Night-time lights (NTLs) represent human activities associated with artificial light^88^, human development and economic dynamics^88–91^, and environmental impacts such as light pollution and green gas emissions^92^. For the SDG15 and Multitemporal HFs, we relied on VIIRS v2 NTL data (available at https://eogdata.mines.edu/products/vnl/) from 2012 to 2022. VIIRS NTLs are processed to suppress background noise, solar and lunar contamination, lightning, outliers, and contamination by cloud cover^88,93^. VIIRS lights are susceptible to representing atmospheric glow around bright sources and background noise instead of actual lights^93^. This effect can blend weak signals from nearby electric lights with stronger signals around gas flares or cities. Notably, the highest NTL values in Ecuador’s northeastern Amazon region and sparse areas in Peru correspond to oil and gas flares. Given the difficulty separating the signals from electric lights and gas flares and the relatively small footprint, gas flares were included in the Electric infrastructure layer. Despite this issue and its coarse resolution of 500 m, there was no substitute for its comprehensive coverage of electrical light information while providing updates since 2012.

In scoring the VIIRS v2 NTL data^28^, we employed a linear function that assigned scores from 0 to 10 based on NTL values ranging from 0.5 to 60. Values below 0.5 were excluded to minimize noise that does not reflect actual ground features. The upper threshold of 60 approximated the highest concentrations of NTL values typically observed within cities in the region. NTL values from gas flares and flower plantations requiring nighttime illumination (“summer flowers”) surpassed this threshold.

We incorporated static electrical infrastructure datasets from OSM^40^ and national sources. Upon visual comparison between national datasets and OSM in both countries, we found OSM provided more precise information (polygons of facilities versus approximate point locations), though attributes were less organized. We also found they are similarly complete. Therefore, we used OSM for the SDG15 HF. Table 4 shows the layers included in this study and their OSM tags.

**Table 4:** OSM tags of Electrical Infrastructure datasets.

Dataset	OSM key	OSM values
**Power plants**	power	plant
**Power plants**	plant:source	solar
**Dams**	waterway	dam
**Substations**	power	substation
**Power lines**	power	line
**Power tower**	power	tower
**Wind turbines**	generator:source	Wind

For the Official HF, we included transmission lines (power lines) and point locations of hydropower plants and stations from national sources: MINAM^49^, the Agency for the Regulation and Control of Energy and Non-Renewable Natural Resources in Ecuador (ARCERNNR)^94^ and the Supervising Agency for Investment in Energy and Mining in Peru (Osignermin)^95^.

Power plants, dams, substations, power towers, and wind turbines received a score of 9, while solar, hydroelectric, and power plants received a score of 8.25 and transmission lines were attributed a score of 5. When datasets corresponded to points, we assigned the corresponding score to surrounding pixels up to 50 m to better represent the size of the features.

We did not find adequate information on flooded hydro reservoirs.

#### Oil and Gas infrastructure

Oil and gas extraction has been associated with environmental degradation in ecologically sensitive regions. It includes the impact of constructing infrastructure such as facilities, water and energy supply systems, deforestation, and water and soil contamination^59,96,97^. The presence of oil wells can lead to contamination in their vicinity due to the release of natural gas and oil flow^98^. Additionally, pipelines require clearing forests and vegetation and can serve as conduits for defaunation and spreading invasive species^99^.

This study included point datasets of wells, platforms, pipelines, stations, deposits, refineries, and warehouses. We obtained static layers from national sources: MINAM^49^, Osignermin^95^ and Perupetro^100^ in Peru and the Ministry of Energy and Non-Renewable Natural Resources (MERNNR)^101^.

We attributed a score of 10 to all oil and gas infrastructure. To better represent the size of features, we assigned the score to surrounding pixels up to 500 m for deposits, refineries, 150 m for filling stations, and 50 m for wells. Additionally, we assigned a direct score of 5.25 to pipelines.

This pressure layer excluded gas flares to prevent duplicating information already captured in the Electric Infrastructure pressure layer through nighttime lights. Furthermore, the layer did not include oil spills due to insufficient information regarding the impacted areas.

#### Mining

Mining can contaminate the water, cause noise pollution, trigger deforestation^102–104^, and affect biodiversity^105–107^.

We incorporated static mining information from official datasets: IGM^39^ in Ecuador and MINAM^50^ in Peru, in addition to OSM^40^, a global compilation of mining data from Maus, V. *et al*.^108^ and multitemporal information from MapBiomas in Peru^31^. Datasets included points and polygons. Typically, mining areas comprise a mix of infrastructure, extraction, and bare soil areas, although no infrastructure or contamination datasets were found.

All mining areas were uniformly assigned a score of 7.25. For point data, we allocated this score to the pixel locations and their immediate surroundings up to 50 m.

We have not differentiated scoring according to mining types or scale. Also, mining datasets have been reported to underrepresent reality^109^.

## Data Records

The 30 m historical series of HF maps for Peru and Ecuador is available on Figshare^110^ at 10.6084/m9.figshare.30226402. Please refer to the Downloading guidance file for download instructions. The **recommended HF version—SDG15—**is shared alongside individual pressure layers, enabling deeper insight and flexible scoring. GIF-relevant HF versions for 2018 (Official and Multitemporal) are also provided, but are not recommended beyond comparisons of input selection strategies^9^. Validation points for Peru and Ecuador (Validation_Points_PE.7z) are available as geopackages, projected to WGS84 UTM 18S (Peru) and 17S (Ecuador). SDG15-, Multitemporal-, and Official-HFs are shown in Fig. 2.

## Technical Validation

We validated the HF maps by calculating agreement and error metrics, comparing them to a higher-quality independent index from the visual interpretation of plots^7^. All versions of the HF maps were validated identically.

The visual interpretation and validation used sample locations generated by the AcATaMa plugin^111^ in QGIS using a stratified random sampling strategy. For strata, we reclassified the Global HF^7^ (GHF) into different levels of human influence: very low (natural habitat, GHF < 4), low (4 ≤ GHF < 8), medium (8 ≤ GHF < 18) and high (GHF ≤ 18). Areas with less human influence are usually larger than areas with higher human influence, potentially creating a sampling imbalance. We used a compromise allocation strategy by starting with a proportional sample allocation and increasing the size for smaller strata as a compromise between the user and producer/overall accuracy^112^. The overall expected error was 0.0015. We used a sinusoidal projection with a central meridian of −74° to validate Peru and Ecuador simultaneously. At each point location, 300 m square plots (matching the pixel shape) were created for direct scoring, and 5 km buffer circles were used to capture the influence of pressure, representing indirect pressures.

The 300 m plot resolution originated from the initial assumption that we would use land cover layers from the European Space Agency^113^ for Peru (the largest country, ~5:1) and keep this resolution for Ecuador. However, Peru’s MapBiomas layers^31^ became available at 30 m resolution after the completion of the visual interpretation. In agreement with users in both countries, all HF maps were produced at a 30 m resolution. Although this mismatch diminished the confidence in the validation metrics, they were still expected to be useful for accuracy comparison across HF versions. It is worth noting that the maps have been evaluated by local experts in the Peruvian and Ecuadorian ministries of the environment as suitable for national use.

The visual interpretation of plots used World Imagery in ArcGIS Pro^10^ (median year 2018). We assigned a visual score based on the type of pressure. Direct pressures received scores from 0 to 3, depending on the percentage of area coverage (polygons) or occurrences (lines and points) within the 300 m plot. Indirect pressures were given a score of 1 when present in the surroundings of the plot (~5 km buffer). Table 5 outlines specific criteria for visual scoring and corresponding pressures.

**Table 5:** Pressures scoring from visual interpretation.

		Areas (direct pressure)	Lines or points (direct pressure)	Indirect pressure
Visual pressures		Urban areas, crops, pasture, disturbed vegetation, forestry	Roads (paved, unpaved, private), railways, tracks, human dwellings, navigable waterways, infrastructure, Other	Roads, navigable waterways, settlements
Visual scoring criteria		By coverage of the plot	By occurrences within the plot	By occurrence around the plot (~5 km)
Scores	0	0%	0	No
	1	1–12%	1	Yes
	2	13%–50%	2	—
	3	>50%	>2	—

Disturbed vegetation was employed when a disturbance could not be attributed to pasture, crops, or forestry. The infrastructure category did not differentiate between electrical, oil and gas, or mining. In addition to the visual pressures, certainty was evaluated using a Yes/No field, filling “No” if the resolution or cloud coverage did not permit adequate plot interpretation. Although no formal resolution threshold was set, imagery had to clearly distinguish ground features, typically with resolutions finer than 50 cm.

Pressure visual scores were standardized to HF values maximums per pressure. We used the visual pressures to match the datasets used in each pressure of the SDG15, Multitemporal and Official HF maps. Visual pressures were combined using the maximum value within the pressures. We used urban areas and indirect pressure from settlements to approximate population density and electrical infrastructure pressures. Additionally, we used a value of 2 to approximate indirect pressures.

We identified 1754 valid plots in Peru and 529 in Ecuador after excluding uncertain plots and locations dominated by water bodies such as rivers and lakes^36,43^. At each plot, HF scores were extracted from the maps.

Three commonly used metrics were calculated to evaluate the agreement between the HF and visual standardized scores^36,114,115^:

- Cohen’s kappa statistic. It assesses the agreement of two scores, accounting for the agreement occurring by chance. We assumed values matched if their difference was less than 20%.
- Root mean squared error (RMSE). The dimensioned error metric is derived from the differences between datasets (HF and visual scores).
- R^2^. Measures the percentage of the variation explained by a linear model.

The best performance across all metrics was observed in the non-restrictive SDG15 HF versions, characterized by lower RMSE and higher Kappa and R^2^ values per country (Table 6). The RMSEs of approximately 0.09 in Peru and 0.11 in Ecuador indicate that both scores were off by roughly 10%. Kappa was 0.91 in Peru and 0.87 in Ecuador, both of which were considered good. R^2^ scores in both countries exceeded 0.60. RMSE and Kappa performed better in Peru, while R^2^ performed better in Ecuador.

**Table 6:** Validation results of the different versions of HF maps in Peru and Ecuador.

Country	HF version	RMSE*	Kappa**	R^2^
Peru	SDG15	0.09	0.91	0.62
	Multitemporal	0.13	0.76	0.44
	Official	0.17	0.65	0.51
Ecuador	SDG15	0.11	0.87	0.69
	Multitemporal	0.17	0.6	0.57
	Official	0.17	0.5	0.65

*Root mean squared error.

**The agreement tolerance was 20%.

## Usage Notes

HF maps offer a spatially explicit representation of cumulative anthropogenic pressures, supporting monitoring and research related to conservation science, landscape ecology, biodiversity assessment, land-use planning, and sustainability analysis.

The 30 m historical series of SDG15 HF maps covers mainland Peru and Ecuador, excluding the Galápagos Islands in Ecuador, and can be used for all ecosystems or other subnational divisions. It was developed to support SDG15 indicators^5^ and may also contribute to other monitoring frameworks, such as the Convention on Biological Diversity (CBD) and its Kunming-Montreal Global Biodiversity Framework (2022–2030), the High Ambition Coalition for Nature and People, the UNESCO Man and the Biosphere (MAB) Programme, the UN Convention to Combat Desertification (UNCCD), and others.

The maps have a 30 m resolution, matching the predominant LULC layers on which they are based. This resolution supports national and subnational assessments—at the provincial level in Ecuador and the departmental level in Peru—of human influence patterns based on broad land use categories, including agricultural and deforestation expansion. Nevertheless, while still informative, it is less suitable for detecting patterns in smaller areas or for detailed site-specific planning.

In research, HF maps provide a continuous variable relevant for conservation and biodiversity modelling, which can be integrated alongside ecological or biophysical variables^23–25,116,117^. The HF index has also been reclassified into categories to support interpretation and visualization (see examples in Technical Validation and additional references^6,118^). Thresholds and bin numbers are context-specific and should be defined based on each analysis. A generic, landscape-based classification to guide data exploration is provided in Table 7. We have added template symbology files to figshare to support initial exploration (See Data Records section).

**Table 7:** HF Index landscape classification.

HF index range	Landscape general characteristics
[0–1)	Original ecosystem, no significant human influence
[1–4)	Original ecosystem with human influence
[4–15)	Rural landscape—primarily agriculture and pasture, with accessibility and indirect pressures
[15 and above]	Highly modified landscape—mostly urban, dominated by impervious surfaces

The scoring template for Peru and Ecuador’s HF maps retains the global HF approach’s structure, assigning each dataset a score from 0 to a maximum of 10 based on its human influence, and maintaining a threshold of 4 to indicate natural cover loss. This allows for rough comparability between the HF maps for Peru and Ecuador and studies based on the Global HF, such as those that have found thresholds of interest^19,24^. However, thresholds should be determined specifically for Peru and Ecuador, as the scale of analysis differs, and the HF maps rely on country-specific datasets and scoring schemes.

While the HF map validation showed satisfactory overall performance, there were areas for improvement common to cumulative pressure mapping^7,16,43^. Subtle and unobservable pressures, such as hunting, microplastics and climate change effects, are absent or only approximated by the indirect pressure analysis. Additionally, pressures like pollution from oil spills and water reservoirs lacked adequate datasets. Errors from the input datasets will be carried over to the HF maps. The absence of pressure from neighbouring countries might change our assessment of boundary areas. The assumption that pressures should be added (cumulative mapping) neglected non-additive interactions. Furthermore, scoring procedures reflect current field practices, the authors’ knowledge and assumptions, as well as the limitations of the data. Changes in the available inputs and the scoring procedures might alter the results.

In addition to the technical notes above, users may find the following links helpful when working with the HF maps for Peru and Ecuador:

- A Spanish translation of this manuscript is available as a preprint at https://zenodo.org/records/17842350
- The Ministry of Environment of Ecuador hosts its HF-related work at: https://nextcloud.ambiente.gob.ec/index.php/s/qTj2TAnSba5HM7J
- The Ministry of Environment of Peru shares its HF-related work at: https://gis.bosques.gob.pe/portal/apps/storymaps/stories/bc9f4afce8f74c0bb857d3ad661005c6
- The HF dataset is also available through the UN Biodiversity Lab: http://www.unbiodiversitylab.org

## Data Availability

The full dataset is available at 10.6084/m9.figshare.30226402.

## Code availability

The Python code is available for download on GitHub (https://github.com/joluaros/National_Human_Footprint_maps). Note that the scripts require a pre-assembled input database. Users can access the necessary datasets via the links provided in the methods or submit requests where applicable. For guidance on installing the Anaconda environment and executing the scripts, please refer to the Readme file.
